# New Appliances and Things Medical

**Published:** 1909-09-11

**Authors:** 


### NEW APPLIANCES AND THINGS MEDICAL.

[We shall be glad to receive at our Office, 28 & 29 Southampton Street, Strand, London, W.C., from the manufacturers, specimens of all new preparations and appliances.]

### A LIQUID SANITARY SOAP.

It is often very useful in hospital work, and still more in what may be termed extra-hospital work, to have a substance that combines the properties of cleansing, as the good housewife understands it, with those of disinfecting, as the modern surgeon understands it. "Carbolacene," a sample of which has been sent us, and which is made by Messrs. W. and F. Walker, Ltd., of Water Street, Liverpool, claims to combine those advantages. It has been put to severe tests by several eminent men, including Dr. Carl Enoch, the chemist to the Chamber of Commerce of Hamburg, Dr. George Tate, Dr. Edgar Flinn, and Dr. Leon Bertrand. Sir Charles Cameron also mentions that he has used it at Dublin. The substance is made in the liquid form, and dissolves very easily in water. The usual strength is 2½ per cent. A rough and ready proportion recommended by the makers is—a cupful of "Carbolacene" to a bucketful of water. In preparing large quantities for use, one gallon of the liquid soap is used with forty gallons of water. It is claimed that it cleanses and disinfects clothes, carpets, blankets, etc., and it can be used for washing paintwork, floors, also in lavatories, drains, stables, etc., and for washing animals. It also possesses antiseptic properties. It can be employed for washing clothes without the addition of soap, for the combined purpose of thoroughly cleansing and disinfecting at the same time. Dr. George Tate, in his report, states that after submitting it to chemical and bacteriological tests, he finds it both a detergent, a disinfectant, and non-corrosive. Dr. Carl Enoch states that it kills the germs of cholera, typhoid, typhus, and carbuncle, the cholera microbe being killed in five minutes with a 2 per cent. solution. It is sent out in five, ten, and twelve gallon drums, twenty gallon kegs, and forty gallon barrels.

### AN ARTISTIC AND ASEPTIC ENAMEL PAINT.

In the group of enamel paints, called by Messrs. Jenson and Nicholson, of Stratford, London, E., "Robbialac," the makers have endeavoured to produce the same artistic results as were obtained by one of the great artists of the Middle Ages, Della Robbia, by the aid of the modern chemist. The modern construction of hospitals demands that the walls of wards, staircases, and, in fact, of every part of the building, shall afford no home for germs; or, in other words, no home for dust, and that there shall be no cracks in which dust can lodge. As a corollary of this, it is necessary that the wardmaid shall be able to apply a brush vigorously, with copious supplies of water, to every part of the walls without affecting the decorations. Further, modern sentiment demands that in place of the somewhat gruesome patterns that were common upon hospital walls in days gone by, patients shall have something pleasing to look upon. In "Robbialac" all of these objects are attained. The roughest and most imperfectly built wall can have its pores and cracks effectually filled by the preliminary "stopping," the wall being well rubbed down after. The second coat, called by the makers "Matt," which has a certain amount of elasticity, provides a bed for the final coating, "Robbialac" proper, which gives the beautiful glossy enamelled surface that is so pleasing to the eye and so effective against bacilli. It is claimed that "Robbialac" is at least as inexpensive as other paints, one gallon being sufficient to cover 100 sq. yards, at the same labour cost as with ordinary paints. It is made in all standard and art colours, and the makers keep a permanent staff of chemists who are able to produce any new colour that may be desired. It has been used, among other places, for the City of London Lying-in Hospital, for the City of London Guardians' Office, and for his Majesty's new yacht.

### ALLSOPP'S LAGER.

(Messrs. Allsopp and Son, Burton-on-Trent.)

We have received a sample of this lager beer. In the report of The Hospital's Commission on Beer and Stout we gave a full account of the technique of lager-beer brewing, and showed in what way it, and the beer thus produced, differ from ordinary English brewing and English beer. The investigations of the Commission included a visit to Messrs. Allsopp's lager-beer brewery and an examination and analysis of their bottled lager beer. Those who are interested in this subject will do well to read the report in question if they have not already done so. All that it is necessary to say here is that we think lager beer is, from the medical standpoint, to be recommended as a good dietetic beverage. It is lighter in alcohol than ordinary English beer, and yet has a good thirst quenching consistency. We further think that for home consumption—lager beer manufactured in this country is preferable to that imported from abroad. Messrs. Allsopp have succeeded, by great care in the choice of original materials and the greatest attention to brewing technique, in producing a lager beer equal to any imported article and entirely free from all kinds of chemical preservatives. We think this product is thoroughly to be recommended.

